# Bilateral Pheochromocytomas in a Patient with Y175C Von Hippel-Lindau Mutation

**DOI:** 10.1155/2018/8967159

**Published:** 2018-07-10

**Authors:** Olga Astapova, Anindita Biswas, Alessandra DiMauro, Jacob Moalem, Stephen R. Hammes

**Affiliations:** Division of Endocrinology and Metabolism, Department of Medicine, University of Rochester School of Medicine and Dentistry, Rochester, New York 14642, USA

## Abstract

Von Hippel-Lindau (VHL) disease, caused by germline mutations in the* VHL* gene, is characterized by metachronously occurring tumors including pheochromocytoma, renal cell carcinoma (RCC), and hemangioblastoma. Although VHL disease leads to reduced life expectancy, its diagnosis is often missed and tumor screening guidelines are sparse. VHL protein acts as a tumor suppressor by targeting hypoxia-inducible factors (HIFs) for degradation through an oxygen-dependent mechanism.* VHL* mutants with more severely reduced HIF degrading function carry a high risk of RCC, while mutants with preserved HIF degrading capacity do not cause RCC but still lead to other tumors. VHL disease is classified into clinical types (1 and 2A-2C) based on this genotype-phenotype relationship. We report a case of bilateral pheochromocytomas and no other VHL-related tumors in a patient with Y175C VHL and show that this mutant preserves the ability to degrade HIF in normal oxygen conditions but, similar to the wild-type VHL protein, loses its ability to degrade HIF under hypoxic conditions. This study adds to the current understanding of the structure-function relationship of* VHL* mutations, which is important for risk stratification of future tumor development in the patients.

## 1. Introduction

Von Hippel-Lindau (*VHL*) is a tumor suppressor gene associated with inhibition of angiogenesis, apoptosis, cell cycle exit, fibronectin matrix assembly, and proteolysis. Germline mutations in* VHL* occur with a frequency of 1:36,000 in Europe [1] and a 20% de novo rate. Mutations are passed down in an autosomal dominant pattern with almost complete penetrance by the age of 60 [2]. People with* VHL* mutations metachronously develop various benign and malignant tumors. More than half of the patients develop CNS and retinal hemangioblastomas (most commonly in the cerebellum or spinal cord). Other common life-threatening tumors are pheochromocytoma and clear cell renal cell carcinoma (RCC). An increased frequency of pancreatic neuroendocrine tumors, endolymphatic sac tumors, epididymal tumors, and benign cysts in the pancreas and the kidneys is also reported. Because of time lag between occurrences of different tumors, the diagnosis of VHL disease is often delayed, resulting in increased mortality [3].

VHL regulates the cellular response to low oxygen conditions by interacting with hypoxia-inducible factors (HIF*α*: HIF1*α* and HIF2*α*). In normoxic conditions, HIF*α* is hydroxylated on conserved proline residues by prolyl hydroxylases, which require oxygen as a cofactor. Hydroxylated HIF*α* is recognized by VHL which acts in complex with its cofactors elongin B and elongin C to ultimately target HIF*α* for ubiquitination and proteasomal degradation [4]. In hypoxic conditions, prolyl hydroxylases become inactive, and unhydroxylated HIF*α* is not recognized by VHL. This leads to accumulation of HIF*α*, which forms heterodimers that translocate to the nucleus, bind to hypoxia-response elements, and induce the transcription of genes involved in adaptations to hypoxia, including angiogenesis. The same process occurs in the absence of functional VHL and leads to tumorigenesis in patients with VHL disease, most likely through the two-hit model [2, 5].

Studies of over a dozen different* VHL* mutations have identified several phenotypic subtypes revealing a structure-function relationship in which the severity of the mutation predicts the likelihood of RCC [6].* VHL* mutants that retain the ability to downregulate HIF*α* are less likely to be associated with RCC than those that lose that ability [4, 7, 8]. Patients with type 1 VHL have deletion or truncation mutations that completely abolish any functional protein expression. They have a high risk of RCC and also present with both retinal and CNS hemangioblastomas; however, pheochromocytomas are uncommon [9]. Type 2 VHL is characterized by pheochromocytomas and is caused by point mutations with variable degrees of limitation of VHL activity. It is further subdivided into 2A, 2B, and 2C based on the frequency of RCC and hemangioblastomas. RCC appears more frequently in patients with the more severely dysfunctional VHL resulting in higher expression of HIF*α*, such as types 1 and 2B. By contrast, type 2C mutations retain their ability to fully downregulate HIF*α* and present with only pheochromocytomas, indicating that HIF*α*-independent mechanisms are at play in the pathogenesis of VHL-related pheochromocytomas. Some* in vitro *studies suggest that type 2C VHL mutants cause defective fibronectin matrix assembly, while retaining the ability to suppress HIF*α* and stop the growth of RCC cells in culture [10, 11].

## 2. Case Presentation

A 53-year-old man of Puerto-Rican origin presented to the endocrinology clinic after undergoing bilateral adrenalectomy for multifocal pheochromocytomas. He had a prior history of morbid obesity, obstructive sleep apnea, diabetes, and hypertension. He was followed by his primary care physician for persistent hematuria ranging from 3 to 35 red blood cells per high power field on urinalysis, as well as urinary frequency, weak stream, and nocturia three times per night for the previous three years. He had been unable to tolerate an empiric trial of tamsulosin for benign prostate hypertrophy due to orthostatic dizziness. Negative symptoms pertinent to this case include flushing, headaches, sweating, palpitations, anxiety, blurry vision, or dizziness. His family history was notable for death from a myocardial infarction in his father at the age of 57 and an unknown genitourinary cancer in his sister. There was no family history of adrenal tumors, hyperparathyroidism, medullary thyroid cancer, renal cancer, or pituitary tumors. The patient was a smoker with several past attempts at quitting.

**Figure 1:**
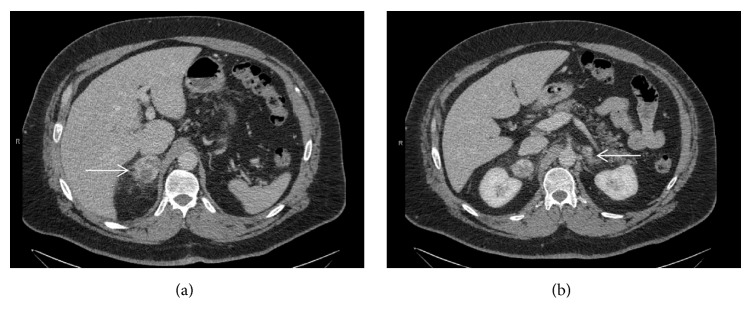
CT scan of the abdomen showing the right adrenal tumor ((a), arrow) and the left adrenal tumor ((b), arrow) prior to adrenalectomy.

Due to the persistent hematuria, smoking, and the family history of cancer, a CT urogram was performed to screen for bladder cancer. While no abnormalities were seen within the urogenital tract, bilateral, irregular, heterogeneous large adrenal masses (Figure 1) measuring 4.7 cm (R) and 1.6 cm (L) were noted. In addition, a prominent and suspicious lymph node was identified. Biochemical characterization of the adrenal masses revealed significantly elevated 24-hour urine normetanephrine (1090 micrograms/gram of creatinine; normal range, 0–400 micrograms/gram of creatinine), leading to the diagnosis of pheochromocytoma. Urine metanephrine level was within normal range. Cushing's syndrome was ruled out with an undetectable late-night salivary cortisol level. Electrolyte levels, kidney function, and complete blood count were within normal limits. In search for additional, extra-adrenal foci, a metaiodobenzylguanidine (MIBG) scan was performed but was nondiagnostic due to lack of cardiac activity. Nevertheless, given the available imaging and biochemical findings, there was concern for malignant pheochromocytoma, and the patient ultimately underwent an open bilateral adrenalectomy and paracaval lymph node excision. Intraoperatively, the patient required vasopressor support and a large amount of crystalloid resuscitation (13 liters) to maintain hemodynamic stability. Intraoperative ultrasound was used to identify one mesenteric lymph node of mildly suspicious appearance which was resected, in addition to a large retroperitoneal paracaval lymph node.

Surgical pathology confirmed pheochromocytomas in the bilateral adrenal glands (right, 5.0 x 3.5 x 2.5 cm, and left, 1.5 x 1.3 x 1.0 cm) which were both confined to the adrenal glands. The paracaval lymph node was described as paraganglioma versus metastatic pheochromocytoma measuring 1.6 cm in the greatest dimension with no lymphoid tissue identified. The immediate postoperative course was unremarkable. The patient was started on life-long glucocorticoid and mineralocorticoid replacement. His diabetes and hypertension resolved.

Due to the multifocal nature of the pheochromocytomas and the presence of first-degree relatives likely to be affected, the patient was offered genetic screening for familial paraganglioma syndromes. With the patient's informed written consent, genomic DNA was isolated from a peripheral blood sample and targeted gene sequencing was performed using PGLNext. Coding exons and adjacent intron nucleotides of the 12 targeted genes associated with hereditary pheochromocytoma syndromes were amplified and then sequenced using PCR and next-generation sequencing. Gross deletion and duplication analysis was also performed. The patient was found to have a heterozygous germline mutation, c.524A>G in the* VHL* gene, corresponding to the Y175C substitution in the protein. This was identified as a likely pathogenic variant and confirmed by Sanger sequencing. In one study, this alteration was described in a patient with a personal and family history of pheochromocytoma and no other VHL-associated tumors and segregated with disease in this family [12].

**Figure 2:**
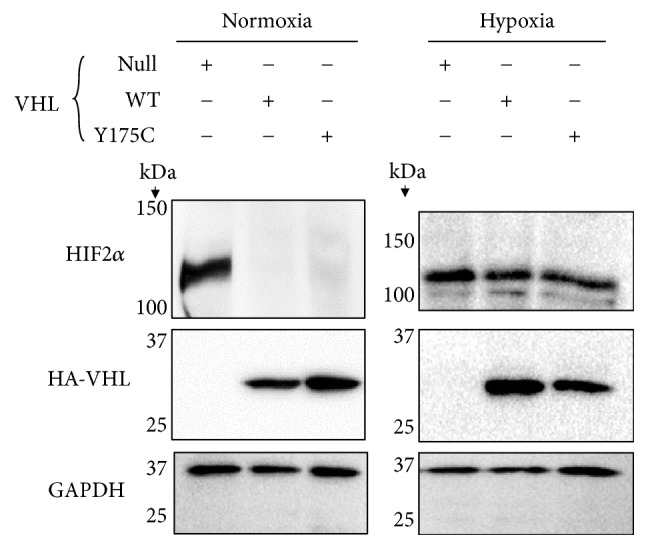
Western blot of HIF2*α*, HA-VHL, and GAPDH expression in normal oxygen (left) and hypoxic (right) conditions. VHL-null 786-O cells were transfected and clonally selected for stable expression of either WT or Y175C VHL or GFP (null), as indicated.

In order to better define the risk of RCC in this patient and others with this mutation, we assessed the ability of Y175C VHL to degrade HIF*α in vitro*. Stable wild-type (WT) or Y175C VHL-expressing cells lines were generated by transfection and clonal selection of* VHL*-null 786-O cells derived from a human RCC as previously described [10, 13]. Control cells were transfected with GFP. We detected HIF2*α* expression in the control* VHL*-null cell line, while stable overexpression of either WT or Y175C VHL resulted in the disappearance of HIF2*ɑ* (Figure 2, left panel). To further characterize the function of Y175C VHL under hypoxic conditions, the cells were placed into a hypoxia incubator at 1% O_2_ for 24 hours. As expected, the WT VHL lost the ability to induce HIF2*α* degradation in hypoxia (Figure 2, right panel). The Y175C VHL similarly did not reduce HIF2*α* abundance in hypoxia. HIF2*α* abundance was also similar in WT and Y175C VHL-expressing cells after 6 hours and 12 hours of hypoxia (data not shown). Thus, under both normoxic and hypoxic conditions, Y175C VHL functions similarly to the WT with regard to HIF*α* degradation.

## 3. Discussion

Differential diagnoses of VHL include other hereditary syndromes such as multiple endocrine neoplasia type 2, polycystic kidney disease, type 1 neurofibromatosis, and hereditary pheochromocytoma-paraganglioma syndrome. Germline mutations in known susceptibility genes including* SDHB* (succinate dehydrogenase complex B) and others are identified in 11-13% of patients with sporadic pheochromocytomas [14]. While screening should not be offered to every patient with a pheochromocytoma, guidelines including those from the Massachusetts General Hospital [1] suggest consideration of genetic screening for syndromes of pheochromocytoma in patients with other factors such as certain other tumors, pancreatic cysts, and multiple pheochromocytomas or those under 40 years of age. In general, genetic screening is likely underutilized according to these guidelines. Genetic screening was recommended for this patient due to the presence of bilateral pheochromocytomas.

Although Y175C VHL has been reported in another family with a similar phenotype, its molecular function has not been studied to date. We have shown that the Y175C mutation preserves the ability of VHL to degrade HIF*α* under normal oxygen conditions. The mutant also functions similarly to the wild-type protein in hypoxia, which abrogates VHL-mediated HIF*α* degradation. This is the first reported molecular study of Y175C VHL and it adds to the growing body of knowledge about various* VHL* mutants. Based on our findings, this patient has type 2C VHL, the subtype with preserved HIF*α* degradation ability and with pheochromocytomas as the sole presenting feature. Previously reported type 2C VHL mutants with pheochromocytoma as the only notable disease manifestation include L188V and V84L. These mutants also demonstrated preserved ability to ubiquitinate HIF*α* [10].

Our case is similar to a previously described Spanish cohort with the same mutation [12] that presented with pheochromocytomas in mutation carriers and no other VHL-associated tumors. The authors of that study calculated the folding energy of Y175C VHL and found that it was only slightly higher than that of the wild-type, indicating that Y175C VHL is predicted to be fairly stable. In contrast, the folding energies of mutants with more severe disease phenotypes are dramatically higher than the wild-type, resulting in unstable proteins and predicted loss of function. Our* in vitro* findings are in line with this* in silico* prediction which supports a low risk of RCC in this patient.

Although HIF*α* degradation is better characterized, many studies have shown that VHL is also important in extracellular matrix assembly and cell membrane structure through regulating fibronectin and integrins, and loss of this function leads to tumor development as well. Fibronectin is upregulated by VHL at the mRNA level and independently of hypoxia [15] or the HIF*α* pathway [16] and requires covalent modification of VHL by NEDD8, a ubiquitin-like molecule [16]. Mutants that escape this modification are involved in tumorigenesis despite adequate HIF*α* suppression. VHL also reduces the protein abundance of several subtypes of integrins in an oxygen-independent manner, via proteasomal degradation, suggesting that VHL is important in cell adhesion and maintenance of tight junctions [17]. Further, depletion of HIF-2*α* alone does not fully recapitulate the effects of VHL replacement in a* VHL*-null cell line [18], particularly the downregulation of integrins and resulting morphological changes. These mechanisms are likely involved in VHL-associated tumorigenesis, particularly in pheochromocytoma, where the HIF*α* pathway does not play a significant role.
